# Phenotypes, Functions, and Clinical Relevance of Regulatory B Cells in Cancer

**DOI:** 10.3389/fimmu.2020.582657

**Published:** 2020-10-22

**Authors:** Jin Shang, Haoran Zha, Yufa Sun

**Affiliations:** ^1^Department of Health Service, Guard Bureau of the Joint Staff Department, Central Military Commission of PLA, Beijing, China; ^2^Department of Oncology, PLA Rocket Force Characteristic Medical Center, Beijing, China

**Keywords:** regulatory B cell, tumor immunology, tumor microenvironment, cancer progression, immunotherapy

## Abstract

In immune system, B cells are classically positive modulators that regulate inflammation and immune responses. Regulatory B cells (Bregs) are a subset of B cells which play crucial roles in various conditions, including infection, allergies, autoimmune diseases, transplantation, and tumors. Until now, unequivocal surface markers for Bregs still lack consensus, although numerous Breg subsets have been identified. Generally, Bregs exert their immunoregulatory functions mainly through cytokine secretion and intercellular contact. In the tumor microenvironment, Bregs suppress effector T cells, induce regulatory T cells and target other tumor-infiltrating immune cells, such as myeloid-derived suppressor cells, natural killer cells and macrophages, to hamper anti-tumor immunity. Meanwhile, the cross-regulations between Bregs and tumor cells often result in tumor escape from immunosurveillance. In addition, accumulating evidence suggests that Bregs are closely associated with many clinicopathological factors of cancer patients and might be potential biomarkers for accessing patient survival. Thus, Bregs are potential therapeutic targets for future immunotherapy in cancer patients. In this review, we will discuss the phenotypes, functions, and clinical relevance of Bregs in cancer.

## Introduction

In immune system, B cells are classically recognized as positive modulators to regulate inflammation and immune responses by releasing antibodies and activating T cells through antigen presentation (1–3). Generally, B cells produce antibodies which are a critical part of the host immunity against infection, that can neutralize pathogens, opsonize pathogens for subsequent phagocytosis and mediate antibody-dependent cellular cytotoxicity (4–6). Besides, B cells also act to suppress immune responses. Accumulating studies have revealed that an immunoregulatory subset of B cells exists and exerts multifaceted functions (7–10). In the 1970s, a suppressive subset of B cells was firstly hypothesized to exist and modulate immune responses, based on reports indicating that the depletion of B cells in splenocytes was correlated with increased severity and duration of delayed-type hypersensitivity in a guinea pig model (11, 12). In the 1980s, it was reported that activated splenic B cells prompted T cells to differentiate into suppressor T cells following adoptive transfer into naïve recipient mice (13, 14), further supporting the concept of “suppressor B cells”. The term “regulatory B cells” (Bregs) was firstly coined in 2002 by Mizoguchi and his colleagues (15). They demonstrated that the interleukin-10 (IL-10)-producing CD1d^+^ Bregs were induced in a chronic inflammatory environment and dampened the progression of intestinal inflammation by repressing inflammatory cascades associated with IL-1 upregulation and STAT3 activation. Thereafter, mounting evidence has uncovered the roles of Bregs in numerous diseases and conditions, such as infection (16), allergies (17), autoimmune diseases (18), transplantation (19), and tumors (20).

To support tumor progression, tumor cells usually generate a tumor microenvironment (TME), which comprises immune cells, fibroblasts, endothelial cells, and the extracellular matrix (ECM). Among the various tumor-infiltrating immune cells in the TME, Bregs release anti-inflammatory mediators and express inhibitory molecules to exert immunoregulatory functions and shape the tumor immune milieu (21–25). In this review, we will discuss the phenotypes, functions and clinical relevance of Bregs in cancer.

## Phenotypes and Immunoregulatory Functions of Bregs

Until now, the definition and classification of Bregs remain unclear due the lack of unique surface markers. As cell populations can be classified based on cytokine production (26), Breg cells are also classified according to their secreted cytokines in many cases and comprise an assortment of subsets (27). Moreover, the phenotypes of human Bregs are not identical to those of murine Bregs. Thus far, several human Breg subsets have been identified. Human CD19^+^CD25^hi^ Bregs, which enhance Treg function, have been reported (28). IL-10-expressing CD24^+^CD38^+^ Bregs were characterized in gastric cancer (GC) patients (29). IL-21-induced CD19^+^CD38^+^CD1d^+^IgM^+^CD147^+^ Bregs, which express granzyme B (GrB), have also been identified in solid tumors (30). In addition, CD19^+^CD24^+^CD38^+^ Bregs were found in invasive breast carcinoma (IBCa) patients, and they were recognized in higher percentages in the breast tissue and peripheral blood of IBCa patients than those in benign tumors and healthy individuals (31). Similarly, well-founded evidence has also revealed several subsets of murine Bregs with different phenotypes. In a B16-F10 melanoma murine model, transitional 2 marginal zone precursor (T2-MZP) Bregs were identified with the B220^+^CD23^+^IgM^hi^CD21^hi^ phenotype in tumor-draining lymph nodes (TDLNs) (32). In another study, splenic CD1d^hi^CD5^+^ Bregs sorted from wild-type (WT) and CD20-deficient mice were adoptively transferred into *Cd19^-^/^-^* mice and CD20 monoclonal antibody (mAb)-treated mice, respectively, resulting in normalized contact hypersensitivity (33). Moreover, it has been determined that IgM^hi^CD1d^hi^CD5^+^CD19^hi^CD23^low^CD38^hi^B220^hi^ Bregs could differentiate into CD138^+^ plasma cells, which secrete IgM and IgG antibodies (34). CD39^+^CD73^+^ Bregs release adenosine and ameliorate the severity of dextran sulfate sodium salt (DSS)-induced acute colitis (35). Regarding tumor, the tumor-associated Breg phenotypes that have been reported to date are listed in **Table 1**.

**Table 1:** Phenotypes and characteristics of tumor-associated Bregs.

Breg Type	Phenotype	Species	Location	Diseases or disease models	Characteristic	Reference
B10 Breg	CD19^+^CD24^+^CD38^+^	Human	Tumors and PB	Invasive breast cancer	Induce Tregs mediated by PD-L1	(31)
	CD19^+^CD5^+^CD1d^+^	Human	PB	Cervical cancer and cervical intraepithelial neoplasia	Inhibit perforin and GrB production by CD8^+^ T cells through IL-10, correlate with FIGO stages, the lymph node metastasis, the tumor differentiation, HPV infection, and the tumor metastasis	(36)
	CD19^+^CD24^hi^CD38^hi^	Human	Tumors and PB	GC	Inhibit IFN-γ and TNF-α by CD4^+^ Th cells through IL-10, induce Tregs through TGF-β1	(37)
	CD27^+^CD10^-^	Human	Tumors and PB	GC	Decrease IFN-γ, TNF, and IL-17 expression by T cells through IL-10	(38)
GrB^+^ Breg	CD19^+^CD38^+^CD1d^hi^ IgM^+^CD147^+^	Human	Tumors	Breast, ovarian, cervical, colorectal, and prostate carcinomas	IL-21 induced, express GrB, inhibit T cell proliferation	(30)
TIM-1^+^ Breg	CD5^hi^CD24^−^CD27^−/+^CD38^+/hi^	Human	Tumors and PB	HCC	Inhibit proliferation and TNF-α and IFN-γ production of CD8^+^ T cells, correlate with disease stage and poor survival	(39)
PD-1^hi^ Breg	CD5^hi^CD24^−/+^CD27^hi/+^CD38^dim^	Human	Tumors and PB	HCC	Result in decreased number and dysfunction of CD8^+^ T cells through IL-10, correlate with disease stage and early recurrence	(40)
PD-L1^+^ Breg	CD20^+^CD27^-^	Human	PB	Melanoma	Suppress IFN-γ by T cells in a PD-L1-dependent manner	(41)
——	PD-1^-^PD-L^+^CD19^+^	Mouse	Spleen and PB	4T1 breast cancer	Induced by MDSCs, inhibit proliferation and IFN-γ production by T cells	(42)
IgA^+^ Breg	IgA^+^CD19^+^	Mouse	Tumors	Colorectal tumor	Overexpress PD-L1, secrete IL-10 and TGF-β, inhibit proliferation and activation of CD8^+^ T cells	(43)
——	CD1d^hi^CD5^+^	Mouse	Spleen	Burkitt-like lymphoma	Suppress CD20 mAb–induced lymphoma depletion and monocyte activation through IL-10	(44)
——	CD19^+^CD24^hi^CD38^hi^	Human	BM and PB	Multiple myeloma	Reduce NK cell-mediated lysis of MM cells	(45)
T2-MZP Breg	B220^+^CD23^+^IgM^hi^CD21^hi^	Mouse	TDLN	Melanoma	Preferentially accumulate in TDLNs, promote tumor growth in B-cell-deficient mice	(32)
——	CD19^+^IL10^+^	Human	Tumors	TSCC	Increased Bregs predict worse prognosis; induce Tregs	(46)
——	CD39^+^CD73^+^	Human	Tumors and PB	HNSCC	Suppress intracellular BTK and Ca2^+^ influx in effector B cells by secreting adenosine	(47)
——	CD19^+^CD24^+^CD38^+^	Human	Tumors and PB	HCC	Interact with liver cancer cells through the CD40/CD154 signaling pathway	(48)
——	CD19^+^CD24^+^CD38^+^	Human	BM and PB	AML	High frequency of Breg cells may predict poor AML prognosis.	(49)
——	CD19^+^CD24^hi^CD27^+^	Human	Tumors and PB	GC	Inhibit proliferation and IFN-γ production by CD4^+^ T cells	(50)
——	CD19^+^CD81^hi^ CD25^+^	Mouse	Tumors	4T1 adenocarcinoma cells and B16F10 melanoma cells	Induce Tregs and decrease CD8^+^ T cells by TGF-β	(51)
——	CD1d^hi^CD5^+^	Mouse	Tumors	PanIN	Regulated by BTK signaling, secrete IL-10 and IL-35	(52)
Plasmablast	CD19^lo^CD27^hi^	Human	Tumors	Colorectal cancer	Gut-homing, inhibit T-cell IFN-γ and TNF-α expression but not promote Foxp3 expression	(53)

Breg, regulatory B cell; GrB, granzyme B; MDSC, myeloid-derived suppressor cell; MM, multiple myeloma; TDLN, tumor-draining lymph node; HNSCC, head and neck squamous cell carcinoma; HCC, hepatocellular carcinoma; HPV, human papillomavirus; TSCC, tongue squamous cell carcinoma; AML, acute myeloid leukemia; GC, gastric cancer; PB, peripheral blood; BM, bone marrow; BKT, Bruton’s tyrosine kinase; PanIN, pancreatic intra-epithelial neoplasia.

Bregs have been reported to exert immunoregulatory functions mainly through cytokine secretion and intercellular contact. The most commonly studied cytokines produced by Bregs are IL-10, IL-35, and transforming growth factor (TGF)-β. IL-10 has multitudinous inhibitory functions and has been widely recognized as an immunoregulatory factor in various chronic inflammatory diseases (54). B10 cells, which are IL-10-producing Bregs, inhibit CD4^+^ T cell proliferation and pro-inflammatory cytokine production by releasing IL-10 (55–57). During chronic hepatitis B virus (HBV) infection, Bregs repress HBV-specific CD8^+^ T cell responses in an IL-10-dependent manner (58). Moreover, B10 cell-derived IL-10 impairs the functions of dendritic cells and macrophages by inducing tolerant phenotypes (59). Additionally, Bregs inhibit interferon-γ (IFN-γ) produced by NK cells through IL-10 (60). Analogous to IL-10, TGF-β is another pivotal mediator secreted by Bregs to regulate immune responses, and it can induce the generation of regulatory T cells (Tregs) (54, 61). Additionally, TGF-β produced by Bregs augments the expression of cytotoxic T lymphocyte associated antigen-4 (CTLA-4) and Foxp3 in Tregs (28). In non-obese diabetic mice, Bregs induce Th1 cell apoptosis and suppress the activities of antigen-presenting cells *via* TGF-β secretion (62). In autoimmune and infectious diseases, Bregs produce IL-35, which is an IL-12 family member and a crucial negative modulator of T-cell immunity (63). IL-35 produced by Bregs promotes Treg proliferation and impairs Th17 responses to enhance immune tolerance (54). Intriguingly, IL-35 has been found to convert B cells into IL-35-producing Bregs, thus establishing a positive feedback loop (64).

Aside from cytokine secretion, Bregs regulate immune responses through intercellular contact, including ligand-receptor interactions such as CTLA-4/CD86, CD40/CD40L, and Fas/FasL. In a study by Aharon *et al*., a transwell system was used to demonstrate that intercellular contact is the major mechanism through which Bregs increase Foxp3 and CTLA-4 expression on Tregs (28). CD28 and CTLA-4 are generally expressed by T cells and are both ligands for CD80/CD86 (65). In another study, Paul and colleagues observed that when co-cultured with autologous T cells, CD19^+^CD24^hi^CD38^hi^ Bregs decreased the percentage of tumor necrosis factor (TNF)-α^+^ and IFN-γ^+^ CD4^+^ T cells, and blocking antibodies against CD80/CD86 partially reversed this suppression (66). Similarly, the simultaneous blockade of IL-10, CD80, and CD86 inhibited Bregs from suppressing IFN-γ and TNF-α production by CD4^+^ T cells in rheumatoid arthritis patients (67). These results indicate that interactions between Bregs and T cells involving CD80/CD86 mediate the suppression of T cell immunity. The CD40/CD40L signaling pathway plays critical roles in establishing humoral responses and is involved in immune responses to tumors (68). Bregs also regulate effector T cells through CD40/CD40L to induce T cell death and inhibit T cell response to autoantigens (69). In addition, blocking the binding of CD40/CD40L between Bregs and CD4^+^ T cells in two different tumor models caused distinct immune responses in terms of Th1/Th2 differentiation and Treg induction (70). Bregs have been reported to express FasL, which belongs to the TNF protein family and causes apoptosis by binding to its receptor, Fas (71). In the spleen, CD5^+^ Bregs express FasL and induce T cell apoptosis through the interactions of Fas/FasL (72). In another study, lipopolysaccharides-induced CD5^+^CD1d^hi^ Bregs inhibited the proliferation of activated CD4^+^ T cells. After adding anti-FasL antibodies, the suppression of CD4^+^ T cell proliferation was partially reversed, suggesting Fas/FasL pathway-mediated regulation of Bregs (73). CD5 expression on Bregs should also be considered (74). In a B16 melanoma model, it was demonstrated that CD5^+^ B cells bound to IL-6 directly through CD5 to promote tumor growth (75). These studies reveal that intercellular contact is critically important for Bregs to exert their immunoregulatory functions.

## Mechanisms Underlying Breg-Mediated Regulation of Antitumor Responses

**Figure 1:**
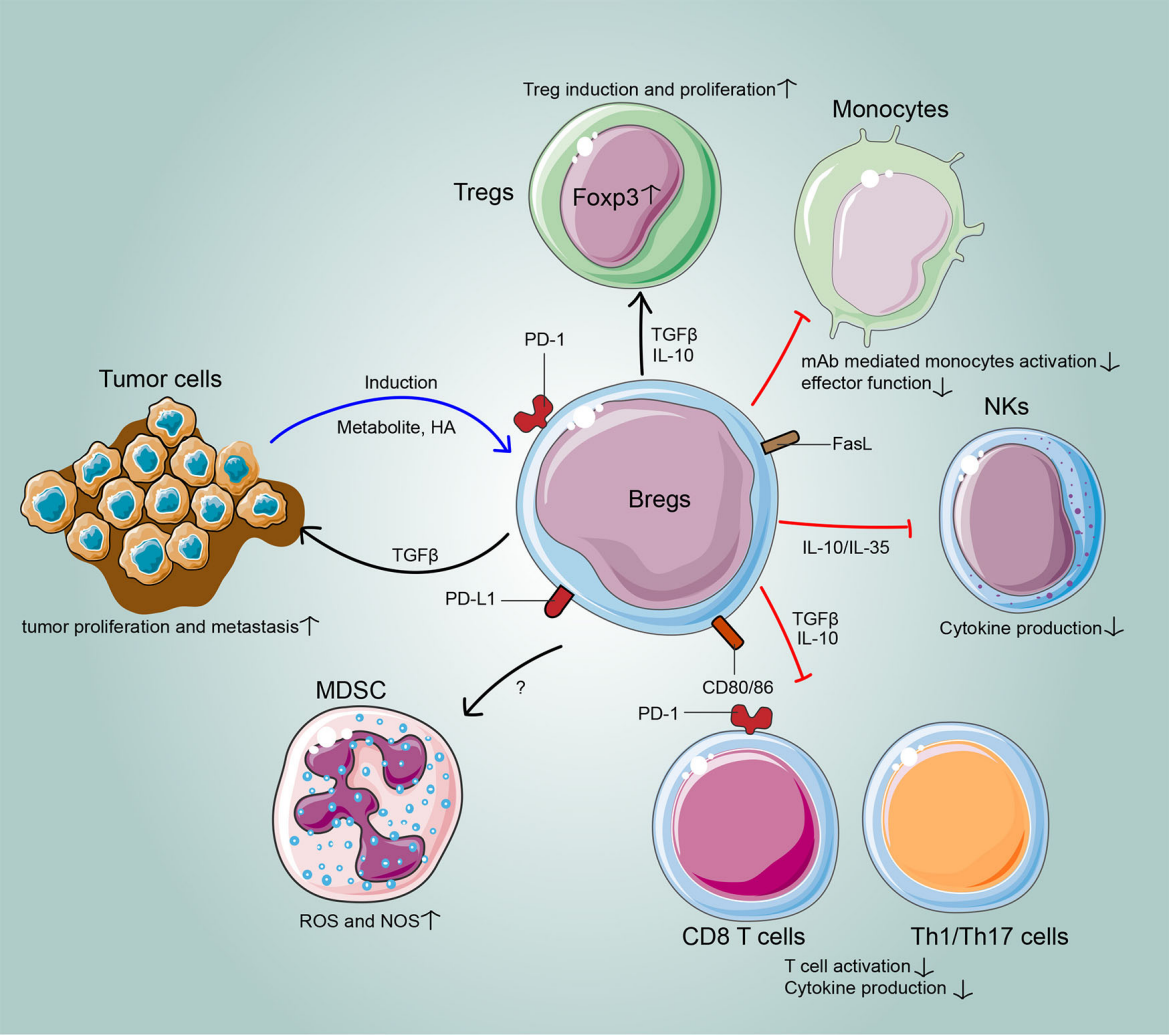
The cross-regulation between Bregs and tumors. In the tumor microenvironment, Bregs suppress effector T cells, induce regulatory T cells and target other tumor-infiltrating immune cells, such as myeloid-derived suppressor cells, natural killer cells and macrophages, to hamper anti-tumor immunity. Meanwhile, the cross-regulations between Bregs and tumor cells often result in tumor escape from immunosurveillance.

The immunoregulatory mechanisms of Bregs have gradually been elucidated in cancer. In the TME, the relationship between the host, tumor, and stroma is mediated by the balance of all tumor-infiltrating cells. Bregs interact with various tumor-infiltrating immune cells of the innate and adaptive immune systems to attenuate anti-tumor responses. Moreover, the cross-regulation between Bregs and tumor cells facilitates tumor progression (**Figure 1**; **Table 2**).

**Table 2:** Targets of Bregs in tumor microenvironment to regulate antitumor responses.

Targets	Cancer types	Mechanisms	References
Effector T cells	Breast, ovarian, cervical, colorectal, prostate, gastric cancer; glioma, glioblastoma, melanoma, hepatocellular carcinoma	Inhibit proliferation and cytokine production of effector T cells	(30, 36–43, 76–79),
Regulatory T cells	Gastric, breast cancer; tongue squamous cell carcinoma	Convert CD4^+^ T cells into Tregs	(31, 37, 46, 80),
MDSCs	4T1 adenocarcinoma cells and B16F10 melanoma	Educate MDSCs to fully evoke regulatory and prometastatic functions of MDSCs	(81)
NK cells	Mouse EL-4 tumor	Secrete IL-10 to inhibit IFN-γ production and tumor elimination by NK cells	(60)
	Multiple myeloma	Abolish NK cell-mediated lysis of multiple myeloma cells	(45)
Effector B cells	Head and neck squamous cell carcinoma	Secrete adenosine to dampen BKT phosphorylation and Ca^2+^ influx in effector B cells	(47)
Monocytes/macrophages	Lymphoma	Secrete IL-10 to suppress mAb-mediated monocyte activation and effector function, resulting in reduced lymphoma depletion	(44)
Breg-tumor cross-regulation	Hepatocellular carcinoma	Bregs directly interacted with liver cancer cells to enhance cancer growth and invasiveness.	(48)
	Multiple myeloma	Multiple myeloma cells inhibited apoptosis of Bregs.	(45)
	Breast cancer	Breast cancer cells produced metabolites of the 5-lipoxygenase pathway to generate Bregs, leading to cancer escape eventually.	(82)
	B16-F10 melanoma	Bregs were restrictedly accumulated in TDLN, which promoted tumor growth after adoptive transfer.	(32)

Treg, regulatory T cell; MDSC, myeloid-derived suppressor cell; NK, natural killer; BKT, Bruton’s tyrosine kinase; TDLN, tumor-draining lymph node.

### Suppression of Effector T Cell Responses

One of the many approaches by which Bregs modulate antitumor response is the direct inhibition of effector T cell responses. Lindner *et al*. found that IL-21-induced human GrB^+^ Bregs inhibited CD4^+^ T-cell proliferation by transporting active GrB to T cells and degrading the T-cell receptor ζ-chain *in vitro* (30).Furthermore, these GrB^+^ Bregs were found to infiltrate numerous human solid tumors, including breast, ovarian, cervical, colorectal, and prostate carcinomas. Subsequent *in vivo* functional assays should be conducted to validate the immunosuppressive properties of GrB^+^ Bregs. In another *in vitro* co-culture system, Bregs sorted from cervical cancer patients secreted IL-10 to decrease the percentage of CD8^+^ T cells, which produced perforin and GrB, whereas the addition of anti-IL-10 antibodies restored the level of these CD8^+^ T cells (36). Similarly, in ovarian cancer patients, B10 cells from ascites significantly lowered the frequency of autologous CD8^+^ T cells secreting IFN-γ (80). In GC patients, depletion of Bregs from peripheral blood mononuclear cells resulted in increased frequencies of IFN-γ^+^ and TNF-α^+^ CD4^+^ T cells (37). Another study identified CD27^+^CD10^−^ Bregs in both peripheral blood and tumor tissues of GC patients (38). Co-culture of these CD27^+^CD10^−^ B cells and autologous T cells showed that IL-10 secretion by CD27^+^CD10^−^ B cells decreased IFN-γ, TNF and IL-17 production by CD4^+^ T cells and IFN-γ and TNF production by CD8^+^ T cells. In addition, TGF-β^+^ Bregs induced by glioma cells inhibited the proliferation and release of perforin and GrB of CD8^+^ T cells (81). In human hepatocellular carcinoma (HCC), TIM-1^+^ Breg cells significantly suppressed the survival and TNF-α and IFN-γ production of CD8^+^ effector T cells (39). Furthermore, Bregs harvested from the glioblastoma tissue of patients suppressed CD8^+^ T cell proliferation and the acquisition of an effector phenotype (82). Moreover, PD-L1^+^ Bregs from stage II/III/IV melanoma patients impaired IFN-γ production by CD8^+^ T cells in a PD-L1-dependent manner in a co-culture system (41). Another study by Xiao *et al*. demonstrated a novel protumorigenic PD-1^hi^ Breg subset in human HCC (40). In corresponding tumor-bearing mice, these PD-1^hi^ Bregs mediated the reduction and dysfunction of CD8^+^ T cells after triggering PD-1 in an IL-10-dependent manner. Moreover, Toll-like receptor-4-mediated upregulation of BCL6 was involved in inducing PD-1^hi^ Breg in the HCC microenvironment. The PD-1/PD-L1 signaling pathway in T cells has been extensively explored, but its role in B cells has received less attention. This study reveals the inhibitory role of PD-1/PD-L1 signaling in Breg-mediated immunosuppression in HCC. Thus, the mechanism underlying clinical tumor regression achieved by anti-PD-1 and anti-PD-L1 antibodies might also involve blocking PD-1/PD-L1 signaling on Bregs.

In a murine breast cancer model, a novel PD-1^-^PD-L1^+^CD19^+^ Breg subset from 4T1-bearing mice exerted the greatest suppressive function on the proliferation and IFN-γ production of T cell in an established B cell/T cell co-culture system (42). These results confirm the role of PD-1/PD-L1 as an immune checkpoint involved in the suppressive properties of Bregs in tumor models. Further studies could investigate whether PD-1^+^/PD-L1^+^ Bregs exert dominant suppressive functions in HCC or breast cancer. If so, then B-cell depletion might restore antitumor responses. In murine colorectal tumors, IgA^+^ Bregs expressed high quantities of immunoregulatory molecules (PD-L1, IL-10, and TGF-β) and suppressed the proliferation and activation of CD8^+^ T cells (43). Moreover, the EMT-6 murine mammary adenocarcinoma cells stimulated splenic B cells to differentiate into Bregs *in vitro*, which impaired the proliferation and IFN-γ production of effector T cells (76).

### Enhancement of Treg Induction

Aside from directly regulating effector T cell responses, Bregs also induce and promote Tregs to create an immunosuppressive microenvironment. In GC patients, Bregs upregulated Foxp3 expression in CD4^+^CD25^-^ effector T cells in a TGF-β1-dependent manner (37). Similarly, Bregs from IBCa patients induced more Treg production than did Bregs from healthy individuals *in vitro* (31). In terms of tongue squamous cell carcinoma (TSCC), Bregs co-cultured with a TSCC cell line converted CD4^+^CD25^-^ T cells into Tregs (46). In a mouse 4T1 model of breast cancer, tumor-evoked Bregs (tBregs) transformed resting CD4^+^ T cells into Foxp3^+^ Tregs by secreting TGF-β to promote lung metastases (77). Moreover, Guan *et al*. found that PD-L1^hi^ breast cancer cell lines stimulated CD19^+^ B cells to form Bregs, which subsequently induced Tregs *in vitro* (31). These studies revealed that both human and murine Bregs could induce Tregs in the TME, and the mechanism underlying these Treg induction requires further investigation to allow for possible disruption of the link between tumor Bregs and Tregs.

### Bregs and Myeloid-Derived Suppressor Cells (MDSCs)

MDSCs are a group of immature cells that are potent in immune suppressors in cancer (83–85). The expansion of MDSCs has often been recognized as an indicator of tumor burden and metastasis (86, 87). However, Bodogai *et al*. observed that B-cell depletion significantly dampened the production of reactive oxygen species and NO by MDSCs as well as the suppressive effect of MDSCs on the proliferation and production of GrB and IFN-γ of CD8^+^ T cells, while adoptive transfer of tBregs restored MDSC-mediated suppression of T cells in a B-cell-deficient mouse model, thus promoting cancer escape and metastasis (78). And experiment results are consistent in other tumor models and human cells. Their study demonstrates that tBregs educate MDSCs to fully evoke the regulatory and prometastatic functions of MDSCs, and cancer-stimulated expansion of MDSCs is not necessarily related to their regulatory functions.

### Other Tumor-Infiltrating Targets of Bregs

Natural killer (NK) cells are critical effectors of the host innate immune system, and they can directly lyse pathogen-infected and injured cells (88). NK cells have been determined to participate in caner immunology (89, 90). Using a B-cell knockout mice model, Inoue and colleagues demonstrated that EL-4 gag tumor cells stimulated B cells to secrete IL-10, which in turn inhibited IFN-γ production and tumor elimination by NK cells (60). In patients with hematological malignancies, bone marrow-derived Bregs abolished NK cell-mediated lysis of multiple myeloma (MM) cells *in vitro* (45). Regarding tumor-infiltrating B cells, Bregs from head and neck squamous cell carcinoma (HNSCC) patients produced adenosine to dampen the phosphorylation of Bruton’s tyrosine kinase (BTK) and Ca^2+^ influx in effector B cells; thus adenosine signaling may be a possible therapeutic target in HNSCC (47). Until now, the effects of Bregs on tumor-infiltrating monocytes/macrophages remain mostly uninvestigated. In a lymphoma mouse model treated with anti-CD20 mAbs, Bregs produced IL-10 to suppress mAb-mediated monocyte activation and effector function, resulting in reduced depletion of lymphoma cells (44).

### Cross-Regulation Between Bregs and Tumor Cells

In addition to infiltrating immune cells, Bregs also directly interacted with liver cancer cells through the CD40/CD154 signaling pathway to enhance HCC growth and invasion (48), indicating that disruption of tumor-Breg interactions might be a potential therapeutic strategy to treat HCC. Moreover, Bregs adoptively transferred into B-cell-deficient mice rescued the growth of *Kras*-expressing pancreatic ductal epithelial cells by secreting IL-35, indicating the role of Bregs in carcinogenesis (91). Intriguingly, tumor cells also induce the generation of Bregs to suppress antitumor immunity. In MM patients, Breg survival was enhanced through MM cell-mediated inhibition of Breg apoptosis in the bone marrow (45). Similarly, breast cancer cells produced metabolites of the 5-lipoxygenase pathway to activate the peroxisome proliferator-activated receptor α (PPARα) in B cells, resulting in tBreg generation; unsurprisingly, inactivation of PPARα prevented tBreg-mediated cancer escape (79). In mice bearing B16-F10 melanoma, T2-MZP Bregs were specifically accumulated in TDLNs (32). Adoptive transfer of these Bregs into B-cell-deficient mice promoted tumor growth, which was not mediated by IL-10 secretion. A deeper understanding of the mechanisms underlying the preferential accumulation of T2-MZP Bregs and promotion of tumors might benefit therapeutic strategies for cancer. The findings discussed above reveal that Bregs and tumors interact and regulate each other in the TME.

## Potential of Bregs as Biomarkers and Prognostic Factors for Cancer

Bregs have been confirmed to be associated with the clinicopathological characteristics of tumors and correlated with the prognosis of cancer patients (**Table 3**).

**Table 3:** Clinical relevance of tumor-associated Bregs.

Reference	Breg Types	Cancer Type	Patient Number	Significant Correlation with Clinicopathological Features	Prognostic Significance
(48)	Circulating Bregs	HCC	74	Tumor UICC stages, tumor multiplicity and venous infiltration	——
(36)	Circulating Bregs	Cervical cancer	70	FIGO stages, lymph node metastasis, tumor differentiation, HPV infection and tumor metastasis	——
(39)	Tumoral Bregs	HCC	51	TNM stage, microvascular invasion and early recurrence	Negatively correlated with DFS and OS of patients who underwent curative surgical resection
(46)	Tumoral Bregs	TSCC	46	Correlated with clinical stage, local recurrence, and regional recurrence	Negatively associated with OS of TSCC patients
(49)	Bregs in PB and BM	AML	46	——	An increased Breg percentage indicated a shorter OS for older patients or patients with high WBC levels.
(50)	Tumoral Bregs	GC	30	——	Percentage of Bregs in tumor tissues was an independent prognostic indicator of GC patient survival.
(40)	Tumor Bregs	HCC	43	——	Frequencies of PD-1^hi^ Bregs in tumor tissues were significantly correlated with disease progression in patients.

Breg, regulatory B cell; HCC, hepatocellular carcinoma; UICC, Union for International Cancer Control; FIGO, International Federation of Gynecology and Obstetrics; HPV, human papillomavirus; DFS, disease-free survival; OS, overall survival; TSCC, tongue squamous cell carcinoma; AML, acute myeloid leukemia; WBC, white blood cell; GC, gastric cancer; PB, peripheral blood; BM, bone marrow.

In TSCC, immunohistochemical staining of Bregs was performed on the paraffin-embedded tissue sections of 46 TSCC patients (46). The results showed that the percentage of Bregs was significantly correlated with clinical stage, local recurrence, and regional recurrence (*P*< 0.05). Moreover, Kaplan–Meier analysis showed that an increased Breg frequency predicted significantly worse overall survival (OS) of TSCC patients. Regarding hematological malignancy, Lv *et al*. investigated the effects of age, white blood cell (WBC) level and Breg frequency on the survival of acute myeloid leukemia (AML) patients (49). They found that an increased Breg percentage indicated a shorter OS for older patients or patients with high WBC levels. Additionally, the frequency of circulating Bregs was significantly correlated with FIGO stages, lymph node metastasis, tumor differentiation, human papillomavirus (HPV) infection, and the tumor metastasis of cervical cancer (*P*<0.05), and this frequency decreased significantly after radical resection of cervical cancer (36). Thus, Bregs may also act as an indicator when evaluating cervical cancer development. Moreover, Yuki and colleagues divided GC patients into Breg^Low^ and Breg^High^ groups based on Breg frequencies in tumor tissue. Survival analysis showed that five-year OS rates in the Breg^Low^ group were significantly higher than those in the Breg^High^ group. Multivariate analysis revealed that the percentage of Bregs in tumor tissue was an independent prognostic indicator of GC patient survival (50). This study indicates that Breg-related immunosuppression is closely correlated to tumor progression. In clinics, disseminated micrometastases overlooked by ordinary diagnostics are a likely reason for tumor recurrence. We propose that sustained Breg-mediated immunosuppression creates an ideal environment for residual cancer cells to grow and develop, eventually leading to recurrence.

In HCC patients, the frequency of TIM-1^+^ Bregs in the tumor tissue was positively associated with patient TNM stage, microvascular invasion and early recurrence. Additionally, Kaplan-Meier analysis verified that the density of tumor-infiltrating TIM-1^+^ Bregs was negatively correlated with disease-free survival (DFS) (n = 101, *P*=0.018) and OS (n = 101, *P*=0.007) of patients who underwent curative surgical resection (39). The results suggest that TIM-1^+^ Bregs could serve as a potential indicator when evaluating tumor progression and making clinical decisions in HCC. In another study by Shao *et al*., peripheral blood samples from 21 normal individuals and 74 HCC patients who underwent hepatectomy were examined (48). The results showed that the frequency of circulating Bregs was significantly correlated with tumor UICC stages (*P*=0.019), tumor multiplicity (*P*=0.023) and venous infiltration (*P*=0.029). Therefore, during the postoperative period, the dynamics of circulating Bregs in HCC patients might be a potential predictor of tumor recurrence. Furthermore, Xiao and colleagues observed that in HCC, the frequencies of PD-1^hi^ Bregs in tumor tissues were significantly associated with disease progression in patients, and 2.6 times more patients with higher frequencies of PD-1^hi^ Bregs displayed early recurrence than those with lower frequencies (40). Interestingly, another study demonstrated that HCC patients with high proportions of tumor-infiltrating B cells showed better prognosis compared with those with low proportions (92), indicating that the existence of B-cell subsets with antitumor functions in tumor tissues. Considering the antitumorigenic and protumorigenic properties of B cells in the TME, we believe that the distribution and subset arrangement of B cells underpin their distinct functions in specific intratumor regions.

## Therapeutic Breg-Targeting Strategies for Cancer Treatment

As Bregs generally exert immunosuppressive and protumorigenic functions, it is noteworthy that Bregs could be potential therapeutic targets of cancer. Several preclinical studies have targeted Bregs in various cancer models. Mitogen/extracellular signal regulated kinase (MEK) is an intermediary component of the mitogen-activated protein kinase (MAPK) pathway. MEK inhibition has shown effects on tumors with MAPK activation both alone and in combination with other targeted therapies (93–95). In a colorectal cancer model *in vivo*, cobimetinib, a MEK inhibitor, decreased the number of Bregs in TDLNs while sparing anti-tumor humoral immunity (96). Mechanistically, MEK inhibition might reduce Bregs through the interruption of chronic BCR signaling, thus impairing the upregulation of specific suppressive surface molecules. Regarding HCC, total glucoside of paeony (TGP), which is extracted from the root of Paeonia Lactiflora, decreased the proportion of B10 cells in the spleens of experimental rats, which at least partially contributed to the anti tumor effect of TGP on rat HCC (97). In addition, lipoxin A4 (LXA4) is an arachidonic acid-derived anti-inflammatory lipid mediator that possesses anti-tumor potential through modulating tumor-immune microenvironments. In tumor-bearing mice, LXA4 suppressed Breg induction, thereby reducing Tregs in draining lymph nodes and tumor tissues as well as augmenting cytotoxic T cell activities (98). Intriguingly, LXA4 targeted Bregs selectively and didn’t affect the proliferation, differentiation and germinal center formation of conventional B cells. Moreover, resveratrol, a plant-derived phytoalexin, inhibits tumor angiogenesis and is a potential anticancer therapeutic drug (99). In mice with highly metastatic mammary 4T1.2 adenocarcinoma, noncytotoxic low doses of resveratrol preferentially dampened tBreg generation and concurrently impaired the tBreg-induced conversion of Foxp3^+^ Tregs to block lung metastasis (51). In pancreatic ductal adenocarcinoma, CD1d^hi^CD5^+^ Bregs exert protumorigenic functions by promoting tumor cell proliferation. Shipra Das and colleagues identified BTK as a vital modulator of CD1d^hi^CD5^+^ Breg differentiation and immunosuppressive function (52). The BTK inhibitor tirabrutinib suppressed CD1d^hi^CD5^+^ Breg differentiation as well as IL-10 and IL-35 secretion *in vitro*. Moreover, tirabrutinib treatment of mice bearing orthotopic Kras^G12D^-pancreatic lesions increased stromal CD8^+^IFN-γ^+^ cytotoxic T cells and attenuated tumor cell proliferation and pancreatic intra-epithelial neoplasia (PanIN) growth. Thus, Bregs may be potential targets of tirabrutinib in PanIN. These studies have confirmed the practicability of inhibiting Bregs to suppress cancer progression. Further investigations are needed to develop a Breg-targeting therapeutic regimen for cancer.

## Challenges and Future Implications

Despite accumulating evidence corroborating the immunoregulatory functions of Bregs in tumor immunology, there are still few unequivocal surface markers for Bregs. Unlike Foxp3 on Tregs, a specific transcriptional factor for identifying Bregs has not been found. One reason for the differences in reported Breg phenotypes may be the various methods utilized to isolate Bregs. It is debatable whether immunoregulatory functions are innate and whether Bregs and their specific signatures are only the results of the adaptation of B cells to different TME stimuli. Lighaam and colleagues reported that *in vitro*-induced human B10 cells lacked specific surface markers, and their IL-10 expression was transient (100). Therefore, the intricate mechanism underlying Breg induction and function during cancer progression needs to be further explicated.

In summary, Bregs have been widely recognized as a subset of B cells that regulate inflammation and antitumor responses. Generally, Bregs exert their functions mainly through cytokine secretion and intercellular contact. In the TME, Bregs suppress effector T cells, induce regulatory T cells and target other tumor-infiltrating immune cells, such as MDSCs, NK cells, and macrophages, to hamper anti-tumor immunity. Meanwhile, the cross-regulations between Bregs and tumor cells often results in tumor escape from immunosurveillance. In addition, Bregs are closely associated with many clinicopathological factors of cancer patients and might predict patient survival. It is imperative that subsequent efforts focus on identifying unique surface markers of Bregs and developing Bregs as potential targets for future Breg-based immunotherapies.

## Author Contributions

JS and HZ prepared the first draft of the manuscript. YS designed and critically revised the manuscript. All authors contributed to the article and approved the submitted version.

## Funding

This work was supported by the National Natural Science Foundation of China under Grant [number 31900627].

## Conflict of Interest

The authors declare that the research was conducted in the absence of any commercial or financial relationships that could be construed as a potential conflict of interest.
